# Svalbard’s 2024 record summer: An early view of Arctic glacier meltdown?

**DOI:** 10.1073/pnas.2503806122

**Published:** 2025-08-18

**Authors:** Thomas Vikhamar Schuler, Rasmus Emil Benestad, Ketil Isaksen, Halfdan Pascal Kierulf, Jack Kohler, Geir Moholdt, Louise Steffensen Schmidt

**Affiliations:** ^a^Department of Geosciences, University of Oslo, Oslo NO-0316, Norway; ^b^Climate and Environment Department, The Norwegian Meteorological Institute, Oslo NO-0313, Norway; ^c^Geodetic Institute, Norwegian Mapping Authority, Hønefoss NO-3507, Norway; ^d^Glaciology and Geology Section, Norwegian Polar Institute, Tromsø NO-9296, Norway

**Keywords:** Arctic land ice, climate change, sea-level rise

## Abstract

A record-breaking melt season affected the Arctic glaciers of Svalbard in summer 2024 by a substantial margin. Across the entire archipelago, glacier melting corresponded to an anomaly of up to four SD and exceeded any previous observation. The pan-Svalbard mass loss in summer 2024 amounts to ~61.7 ± 11.1 Gt and corresponds to 1% of the total ice volume on Svalbard and is comparable to that of the Greenland ice sheet (55 ± 35 Gt), which occupies an area about 50 times larger. Altogether, Svalbard and other glacier regions surrounding the Barents Sea lost 102.1 ± 22.9 Gt of ice in a single year and contributed 0.27 ± 0.06 mm (of which 0.16 mm alone is due to Svalbard) to global sea-level, putting the circum-Barents region among the strongest contributors to global sea-level rise in 2024. Most of the 2024 glacier melt occurred during a 6-wk period of persistent atmospheric circulation pattern causing record-high air temperatures, an event with an extremely low recurrence interval under current climate conditions. However, future climate projections suggest that such temperature levels will become increasingly commonplace by the end of the 21st century, potentially even surpassing those of 2024. Svalbard’s summer of 2024 serves as a forecast for future glacier meltdown in the Arctic, offering a glimpse into conditions 70 y ahead.

## Significance

Affecting global sea-level rise, mass loss from Arctic glaciers has implications far beyond their geographical location and is relevant for related geosciences, as well as ecological and biosciences and the general public. Combining in situ observations, remote sensing, and modeling, we quantify the mass loss of all glaciers on Svalbard during the record-warm summer of 2024, that by far exceeds previous levels. Analyzing historic temperature records and projections of future climate, we find that temperature levels as in 2024 represent a rare situation for contemporary climate conditions but will be frequently reached in a few decades. We suggest that the summer of 2024 in Svalbard serves as an analogue for the widespread meltdown of Arctic glaciers in a warmer world.

The accelerated warming of the Arctic causes widespread glacier mass loss, contributing to global sea-level rise (1, 2) and having potential implications for large-scale ocean circulation like the Atlantic Meridional Overturning Circulation (3, 4). Regionally, glacier melt impacts fjord circulation (5, 6), marine ecology (7, 8), as well as related wildlife and local communities. The majority of the world’s land ice mass, outside the ice sheets, resides in the Arctic, of which less than 10% is in Svalbard (1). Despite a much smaller glacierized area, the sea-level rise contribution of Svalbard in 2024 was comparable to that of the entire Greenland ice sheet (9). Here, we quantify the record mass loss during summer 2024, which corresponds to about 1% of the total ice volume on Svalbard (10), significantly exceeding any previous estimates (11). Most of the record melting happened over 6 wk of persistently high air temperatures, a statistically rare situation for current climate conditions. However, our study shows that 2024 summer temperatures will be frequently reached in just a few decades and exceeded toward the end of the 21st century. The summer of 2024 on Svalbard thus provided a window into Arctic glacier meltdown in a warmer future, highlighting the severe mass loss of glaciers and its repercussions in other regions of the Arctic beyond Svalbard.

## Glacier Mass Balance 2024

**Fig. 1.**
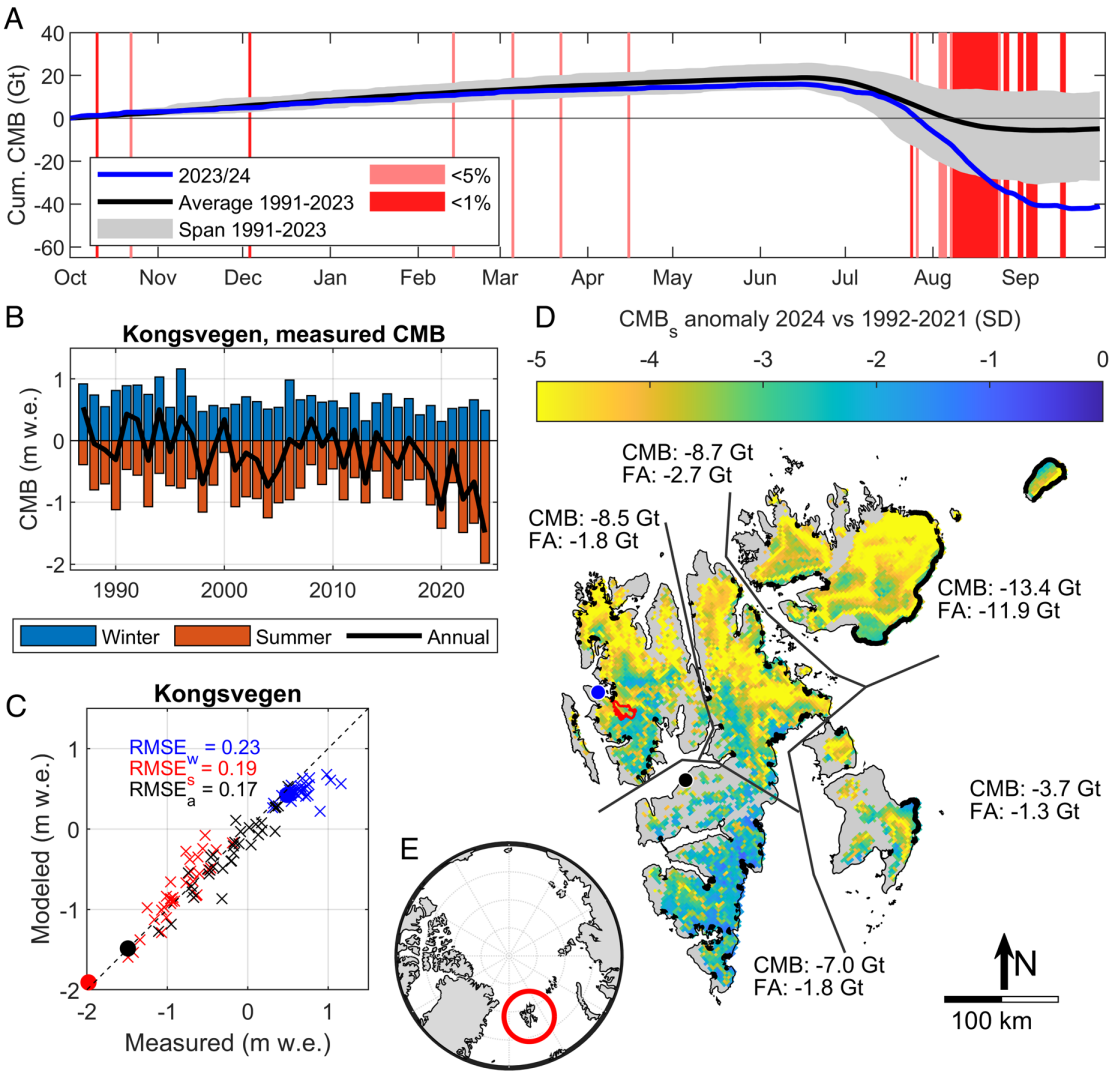
Svalbard glacier mass balance 2024 vs 1991-2020 climatology. (*A*) Cumulative daily CMB for the season 2023/24 (blue) compared to the range of values of previous years 1991–2023 (gray shading) and the average value for the 1991–2023 period (black). Light and dark red shading indicate days where daily CMB was below the 5 and 1% percentile of the 1991–2023 period. (*B*) Seasonal mass balances of Kongsvegen as measured by the glaciological method, for winter (blue) and summer seasons (red) as well as the annual value (black). Values are per unit area and presented in water equivalents (w.e.). (*C*) Comparison of measured and simulated, glacier-wide mass balances of Kongsvegen for the period 1991–2024, again the colors refer to different seasons: winter (blue), summer (red), and annual (black). The values for the extreme year 2024 are shown as filled circles, using the same color code. (*D*) Map of Svalbard in the high Arctic showing color-coded the CMB anomaly for August 2024, relative to the period 1991–2020 and expressed in terms of the climatology SD. The red line shows the boundaries of Kongsvegen glacier. Marine termini are marked with thick, black lines. Labels show estimates of CMB and FA for five subregions (division indicated by lines). Blue and black dots denote locations of the GNSS observatories NYA1 and LYRS, respectively. (*E*) Location of Svalbard (red circle) within the Arctic.

The High-Arctic archipelago of Svalbard (Fig. 1*E*) is recognized as especially exposed to rapid warming, with rates above the already elevated Arctic temperature increase (12). This implies increased mass loss from glaciers (11, 13) that occupy about 53% of the land surface (14). Due to the large area fraction with elevations close to the transition between ablation and accumulation areas, pushing this transition upward may cause major mass loss (15). The total mass balance (TMB) of a glacierized region responds to the exchange of mass and energy between the atmosphere and the glacier, as well as between the ocean and the submarine glacier fronts. The former is termed the climatic mass balance (CMB), and the latter is known as frontal ablation (FA), the sum of frontal melting and ice calving (16). CMB is typically monitored by in situ measurements at individual glaciers, whereas FA usually is determined from remotely sensed information. Ingesting atmospheric forcing, glacier–climate models can simulate the CMB over large spatial and temporal scales. Based on atmospheric reanalysis (17) and weather forecast data (18), the CryoGrid community model (19) computes CMB for all glaciers in Svalbard (20) and the Russian islands surrounding the Barents Sea (21). For Svalbard, daily updated CryoGrid simulations of CMB (Fig. 1*A*) reveal that the mass loss for the glaciological mass balance year Oct 2023–Sep 2024 amounted to 42.1 ± 10.7 Gt and surpassed the 2000–19 estimate (11) by a factor 6, well beyond the previously experienced range since the 1960s (13, 20). While the winter season was very close to the climatological (1991–2020) mean, the significant anomaly occurred during summer, fueled by record melt rates narrowly concentrated in a ~6 wk period between end of July and beginning of September (red shading in Fig. 1*A*).

The anomaly of the glacier mass loss during the summer season (April–September, Fig. 1*D*) was record high all over Svalbard, with the generally milder southern Spitsbergen being less abnormally affected than the colder regions in north and northeast Svalbard (*SI Appendix*, Fig. S1), where levels of up to 5 SD were reached. The spatial mean of this anomaly over the glacier-covered region was 3.6 SD below the 1991–2020 climatology which implies a return interval exceeding 1500 y relative to the statistical distribution of glacier mass balance in the recent past (assuming a normal distribution, *SI Appendix*, Fig. S2). This extreme anomaly is also supported by the long-term, in situ measurements of Kongsvegen glacier (100 km^2^), that show a CMB that was 3.4 SD more negative than the 1991–2020 mean, mainly caused by extremely negative mass changes during summer (Fig. 1*B*). Even though there is uncertainty about the statistics beyond the historical record, we can conclude that the 2024 CMB anomaly had a return period of at least several 100 y.

Analysis of daily CMB revealed that in 2024, 37 d were below the 5% percentile of the 1991–2020 climatology (22 d below the 1% percentile), indicating extensive glacier mass loss. Most of this mass loss happened between 23 July and 8 September, a 46-d period of persistently high melt rates when daily CMB was for 29 d below the 1% percentile of the past 30 y, and for 32 d below the 5% percentile (Fig. 1*A*, *SI Appendix*, Fig. S3). The reliability of the model simulations has been carefully assessed (20) using a range of different glacier measurements and has been confirmed also for the extreme situation in 2024 (Fig. 1*C* and *SI Appendix*, Fig. S4).

To assess the total mass loss, the CMB (−42.1 ± 10.7 Gt) needs to be complemented with estimates of FA of marine-terminating glaciers. Using remote sensing data (22) and a model of ice thickness (10), we estimate a FA of 19.6 ± 3.1 Gt for 2024, of which 3.2 ± 0.5 Gt relates to front area retreat. While this marine ice loss is high, it is not extreme compared to the average for 2010-2020 (16.8 ± 2.5 Gt y^−1^) (23). Together, the total ice loss for the mass balance year 2023/24 amounted to 61.7 ± 11.1 Gt (*SI Appendix*, Table S1), equivalent to about 1% of the total ice volume of all Svalbard glaciers (10).

## Consequences and Perspective

By injecting buoyant freshwater, meltwater runoff from land to the ocean has far-reaching implications for ocean circulation near shore and in fjords (5, 6) and fuels a variety of ecological communities across a wide range of the food chain (7, 8). In 2024, freshwater runoff from Svalbard was heavily affected by the record mass loss and with a total of 72.0 ± 9.4 Gt, more than twice the 1991–2020 levels (*SI Appendix*, Fig. S5*A*).

The sum of persistently high levels of daily runoff between 23rd July and 7th September 2024 resulted in a record-high annual volume according to model simulations. The runoff was above the 99% percentile for 28 out of 46 d and above the 95% percentile for 35 out of 46 d (*SI Appendix*, Fig. S5*B*), closely reflecting similar statistics for CMB (*SI Appendix*, Fig. S3*B*).

The ~42 Gt mass loss by CMB exceeds the loss predicted by 2100 for RCP8.5 (24) by about 70%; however, the latter estimated the average conditions at the end of the 21st century, not the variability around this mean. To put the extreme glacier mass loss during summer 2024 in perspective, we compare the total mass change (−61.7 ± 11.1 Gt) to that of the Greenland ice sheet. Although about 50 times larger in area than the glacier-covered area of Svalbard (1.7 Mio km^2^ vs 32,250 km^2^), the Greenland ice sheet lost about 55 ± 35 Gt in the balance period 2023/24 (9). The 61.7 Gt figure of Svalbard mass loss is slightly below the total mass of Jostedalsbreen (25), the largest glacier system in mainland Norway, and the 42.1 Gt loss by CMB is close to the current mass of all glaciers in Switzerland (26).

Independent support for the extraordinary magnitude of the glacier mass loss in 2024 comes from geodetic measurements of land uplift, continuously conducted at the Svalbard Geodetic Earth Observatories in Ny Ålesund (since 1993) and Longyearbyen (since 2010). During the summer period (1. Jul to 1. Oct) in 2024, surface height in Ny-Ålesund has increased by 16.4 ± 2.0 mm, whereas the corresponding summer signal during the period 2010–2020 is 9.0 ± 2.0 mm. Accounting for a continuous long-term uplift rate of 6.8 mm y^−1^ (27), the uplifts during the 3 mo summer periods amount to 14.7 mm in 2024 and 7.3 mm for 2010–2020 (resp. 11.7 mm and 6.6 mm for Longyearbyen, *SI Appendix*, Fig. S5). This twofold uplift in summer 2024, with respect to the 2010–2020 mean, corresponds to an anomaly of 3.7 SD (resp. 4.2 σ for Longyearbyen). In comparison, the glacier mass loss during summer 2024 was 2.2 times larger than its 2010–2020 mean, representing an anomaly of 3.6 SD (Fig. 1).

Updated CMB simulations for glaciers on other islands surrounding the Barents Sea (21), namely Franz Josef Land and Novaya Zemlya, also show record negative CMB for summer 2024, amounting to −7.8 ± 3.9 Gt and −21.1 ± 6.6 Gt respectively. Together with decadal estimates for FA rates (7.4 ± 3.3 Gt y^−1^ and 4.2 ± 0.9 Gt y^−1^), this amounts to a total mass loss of 40.5 ± 11.8 Gt from glaciers on Franz Josef Land and Novaya Zemlya. The close connection of climatic variations in the Barents region has been recognized before and is due to the strong dependency on sea-ice cover and sea surface temperature in this region (12).

Out of the 102.2 ± 22.9 Gt total mass loss of the circum-Barents glaciers in 2024, 96.6 ± 22.9 Gt contributed to sea-level change, disregarding 5.6 ± 2.9 Gt of submarine ice loss (*cf* Methods: TMB and sea-level contribution). This corresponds to a global sea-level rise contribution of about 0.27 ± 0.06 mm (of which 0.16 ± 0.03 mm alone is due to mass loss from Svalbard) and corresponds to half of the decade-long sea-level contribution of all Arctic glaciers estimated for 2006–15 (1) (*SI Appendix*, Table S1). This puts the circum-Barents region among the strongest contributors to the global sea-level rise (1, 2) in 2024.

## Meteorological Drivers

**Fig. 2.**
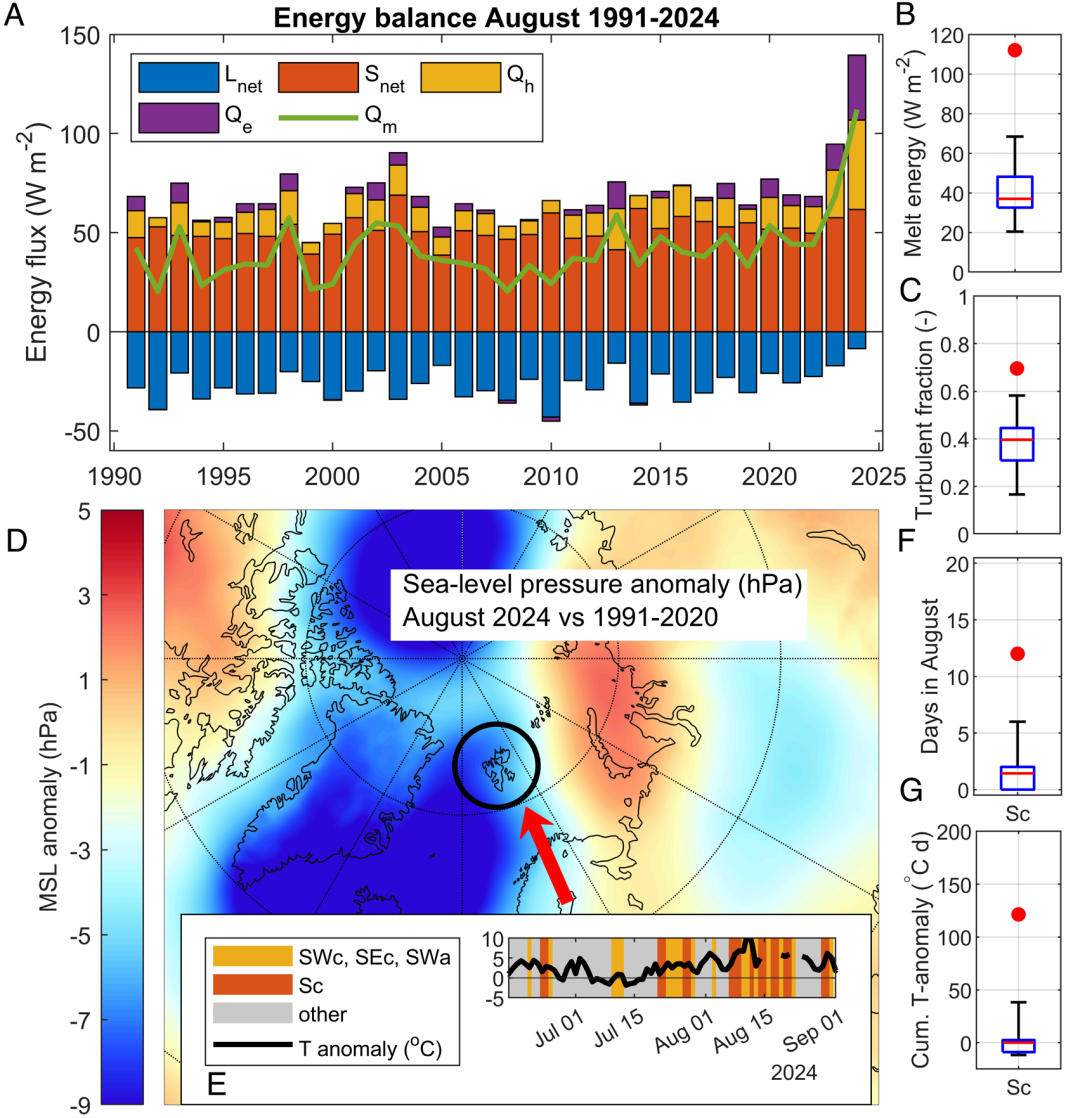
Meteorological drivers of record melting. (*A*) Energy partitioning during August, for each of the years 1991–2024. Q_m_ is the energy flux related to melting of snow and ice, S_net_ is the net shortwave radiative flux, L_net_ is the net longwave radiative flux, Q_h_ is the flux of sensible heat, and Q_e_ the flux of latent heat. The sign convention is such that positive fluxes are directed toward the surface, whereas negative fluxes are directed away from the surface. (*B*) Energy available for melting during August 2024 (red dot) compared to 1991–2023 (boxplot). (*C*) Fraction of melt energy provided by turbulent fluxes during August 2024 (red dot) compared to 1991–2023 (boxplot). (*D*) Sea-level pressure anomaly across the Arctic from ERA-5 (31) during August 2024 relative to the 1991–2020 climatology; the location of Svalbard is indicated by the black circle, the red arrow indicates the direction of airflow. (*E*) Occurrence of southerly circulation types during summer 2024: SWc, SEc, SWa (orange shading) and Sc (red shading), along with observed temperature anomalies at Svalbard Airport (black). (*F*) Occurrence frequency of circulation type Sc in August 2024 (red dot), relative to the climatology 1991–2020 for this month (boxplot). (*G*) Cumulative temperature anomaly for circulation type Sc in August 2024 (red dot), compared to the climatology 1991–2020 for this month (boxplot). The accumulated temperature anomaly accounts for frequency and warming of Sc during August.

To explain the extraordinary glacier mass loss in summer 2024, we examine the surface energy balance during August (Fig. 2*A*). Compared to the climatic period since 1991, the 2024 contributions of sensible (Q_h_) and latent heat (Q_e_) reached record-high levels, accompanied by record low losses by net longwave radiation (L_net_), together resulting in record-high energy available for melting (Q_m_, Fig. 2*B*). While contributions by turbulent heat (Q_h_ + Q_e_) in 1991–2020 usually accounted for about 40% of Q_m_, and rarely exceeded 50%, they were in August 2024 responsible for about 70% of the energy available for melting (Fig. 2*C*). This is indicative of warm air masses advected toward the archipelago. Since the temperature above a melting glacier surface remains relatively low, advection of warm, moist air (large positive Q_h_ contribution) causes condensation (large positive Q_e_ contributions) and high levels of downwelling thermal radiation, giving rise to low longwave losses (small negative magnitude of L_net_), even during long periods of clear weather. Inspecting the August sea-level pressure anomaly (2024 vs 1991–2020) for the Arctic (Fig. 2*D*), a prominent low anomaly is noticeable, centered above Iceland and southern Greenland, accompanied by a positive anomaly east of the Barents Sea. This circulation pattern is also visible at upper levels of the atmosphere (*SI Appendix*, Fig. S9*C*) and is conducive to air advection toward Svalbard from southern directions. This pattern has been dominating over much of the summer period (Fig. 2*E*), much more frequent than usual (Fig. 2*F*) and coincides with periods of large positive temperature anomalies (Fig. 2*E* and *SI Appendix*, Fig. S8). Parts of Northern Europe, recognized as one of the main source areas of the August 2024 air masses in Svalbard (28), had also a record-setting warm summer (29, 30).

A similar pattern has been previously observed in 2013, also then related to a record glacier mass loss (32), albeit much smaller than the 2024 mass loss. Such persistent weather patterns are linked to weaker summer westerlies and a wavier polar jet stream, with increased probability of extreme weather occurrences during the summer months in North America and the Atlantic regions (33, 34), including extensive melt of Arctic land ice (35, 36). Previous analysis has shown that air temperatures in Svalbard are largely determined by the sea-ice extent and the direction of air advection and that the highest temperatures occur when air flows from the southern sector (37). This is also demonstrated by the extraordinarily large cumulative temperature anomaly in 2024 that reached up to 120 °C d, whereas in 1991–2020 it never exceeded 40 °C d (Fig. 2*G*).

**Fig. 3.**
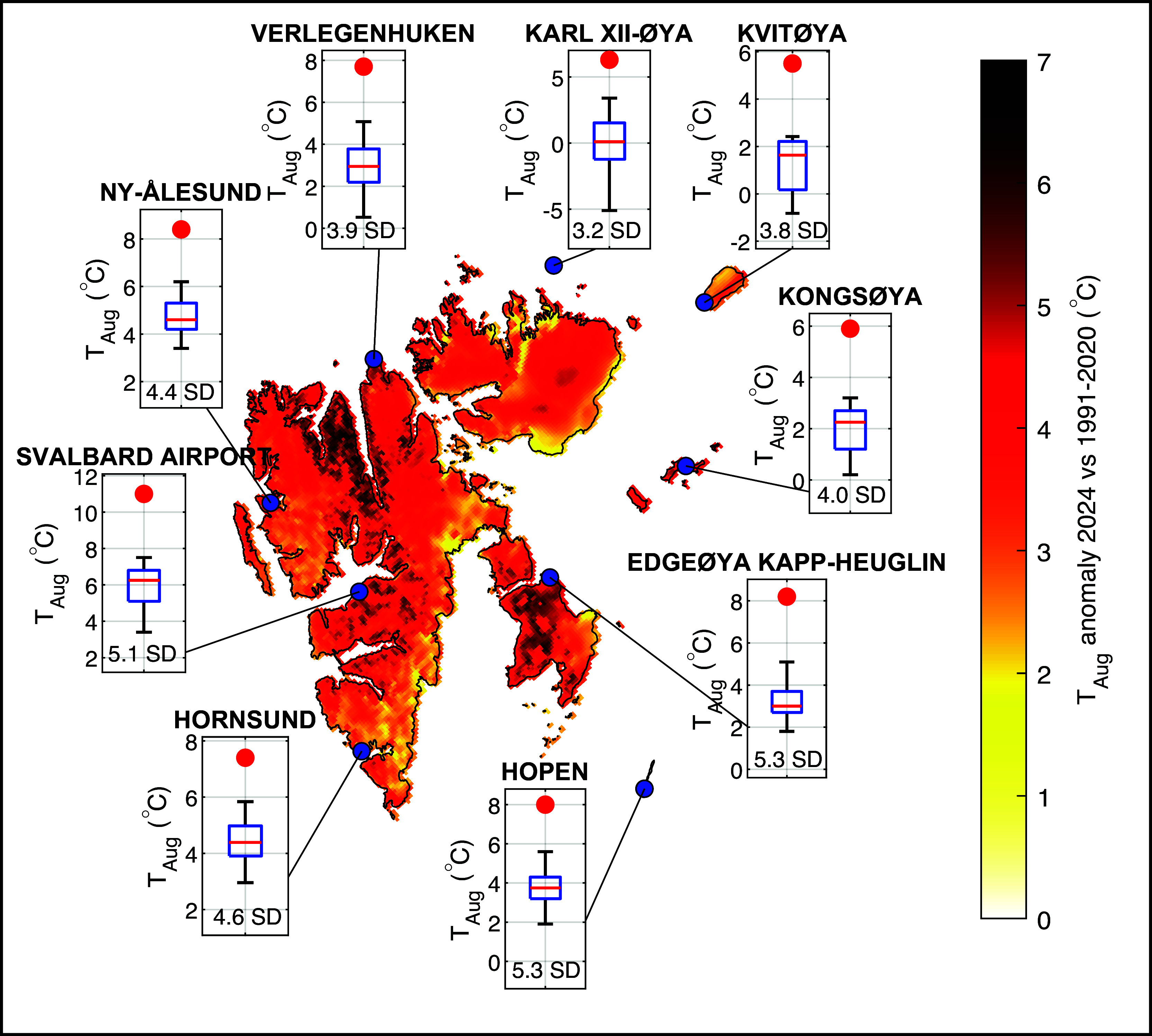
Record air temperature in August 2024. The 2024 anomaly of August air temperature, according to CARRA, with respect to the 1991–2020 climatology. Inserts compare measured temperatures at several long-term, operational weather stations during August 2024 (red dot) with those of the 1991–2020 climatology (boxplot).

As a result of this persistent weather constellation, Svalbard experienced in August 2024 an extraordinarily large temperature anomaly of 3.7 °C (corresponding to a 4.2 SD anomaly in terms of the 1991–2020 climatology) as indicated by reanalysis, weather forecast data, and in situ thermometer readings (Fig. 3). This anomaly was positive everywhere but most intense in northern, inland locations and on Edgeøya, where locally it was more than 6 °C warmer than usual. This model view is corroborated by measurements made at several long-term, operational weather stations around Svalbard (inserts in Fig. 3), all of which reveal anomalies between 3.2 and 5.3 SD. Detailed analysis shows that, for instance, at Svalbard Airport, air temperature exceeded the 99% percentile of the 1991–2020 climatology for 23 out of the 46 d between 23 July and 8 September (*SI Appendix*, Fig. S6).

The high temperatures were paralleled by record-setting specific humidity, measured at weather stations in Svalbard, giving rise to elevated levels of condensation (Q_e_ in Fig. 2*A*). The advected warm and moist air was further amplified by a marine heatwave in late July and most of August in the Barents Sea and northern sections of the Norwegian Sea, where sea surface temperature anomalies were 3.5 to 5 °C above the 1991–2020 baseline (38). The exceptional warmth observed in Svalbard in August was likely a consequence of a mixture of unusually frequent preexisting warm air masses from the south and their interaction with the abnormally warm ocean (28).

## A Window Into the Future?

In the summer of 2024, the anomalous persistence of circulation patterns, superimposed to long-term warming, was responsible for record temperatures and related glacier mass loss at Svalbard. Our simulation reveals that variations in summer CMB are strongly correlated with air temperature (r = −0.78, *SI Appendix*, Fig. S7), and in the absence of detailed projections for glacier evolution, we employ temperature as a proxy to assess the possible evolution of CMB in the future.

A recent study (39) examines the effects of atmospheric circulation variability on the Arctic cryosphere, on top of the general background warming. State-of-the-art global climate models reproduce both the Arctic warming as well as the observed variations in circulation patterns (40), but it is disputed whether or not they adequately represent any long-term trends in the circulation patterns (41, 42). Here, we assume that the climate models continue to simulate representative circulation patterns for the future and show that similar temperature levels as those observed in summer 2024 may be reached by general warming of the atmosphere, without the need of an anomaly situation.

We now study the statistics of projected temperature evolution at Svalbard Airport until 2100, according to downscaled simulations of the Coupled Model Intercomparison Project CMIP6 (43). For each of four Shared Socio-economic Pathways (SSP 1-3 and 5), between 209 and 351 individual simulations by the participating climate models have been downscaled using a well-established empirical–statistical procedure (44).

**Fig. 4.**
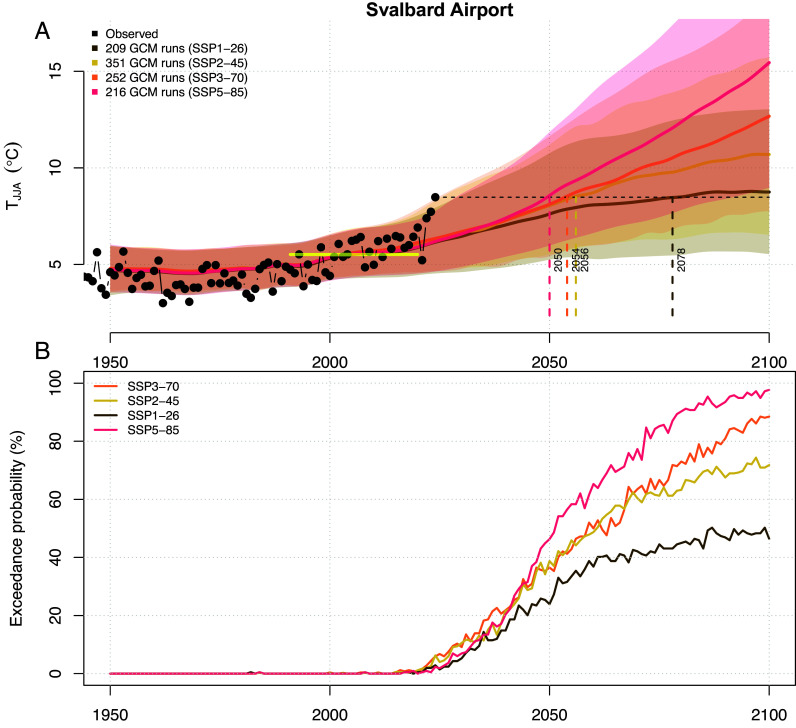
Projected temperature evolution until 2100. (*A*) shows JJA air temperature measured at Svalbard Airport (black) and corresponding downscaled statistics from CMIP6 multimodel ensembles for four different SSPs. The shaded regions mark the 5 to 95% CI for the respective SSP, and the solid lines show the ensemble means. The level of the observed value for 2024 is indicated by the horizontal dashed line, and the yellow horizontal bar indicates the mean value of the 1991–2020 climatology. Vertical bars indicate the time when the ensemble means reach the observed 2024 level. (*B*) shows the fraction of ensemble members with downscaled JJA air temperature exceeding the observed value of 2024, for each of the SSPs.

We use the mean and the 5 to 95% percentile CI to illustrate the ensemble behavior for each SSP from 1950 until 2100, compared to observed values (Fig. 4*A* and *SI Appendix*, Fig. S8). With an average temperature of 8.5 °C in June, July, and August (JJA), 2024 is a very unlikely event even in terms of the ensemble spread for 2024. However, over this 150-y period, the projected summer (JJA) temperatures increase for all SSPs. For the low-emission SSP1-26, temperature starts to stabilize toward 2100 but at an average level corresponding to the summer 2024 anomaly. For the other SSPs, temperature continues rising after 2100, and in the most severe scenario (SSP5-85), the ensemble mean exceeds 15 °C at the end of the century. We also compute the percentage of ensemble members that show JJA temperature exceeding the observed 2024 value. Almost all simulations stay below this value before 2020, after which this percentage starts increasing. For SSP5-85, this percentage exceeds 50% by around 2050 and approaches 100% by 2100, consistent with almost all summers being warmer than that of 2024 if the SSP5-85 emission scenario unfolds. For the SSP1-26 emission scenario, about 50% of the ensemble members give warmer summer conditions by 2100. Since the ensemble spread is comparable to the interannual variability (Fig. 4*A*), these results also suggest that 50% of the future summers will be warmer than 2024, according to the optimistic SSP1-26 emission scenario. This suggests further that the summer of 2024 may represent the normal situation in 2100, and the observed mass loss of glaciers in 2024 indeed provides a view into future glacier meltdown in Svalbard and probably other parts of the Arctic. The observed record-high temperature on Svalbard is consistent with results of recent analysis indicating that the spatial extent of heatwaves has become more widespread both in the Arctic (44) and on a global basis (45, 46).

## Materials and Methods

### CMB from In Situ Observations.

The surface mass balance of Kongsvegen (47) has been measured since 1987 (48) using the glaciological method (16). Aluminum poles are fixed in the ice as reference markers to record the change of glacier surface. Surveys are conducted twice a year, in spring before melting starts and in fall, after the melt season is mostly over. These seasonal measurements allow distinguishing between an accumulation-dominated winter and an ablation-dominated summer balance. To convert the height change into a mass change; a constant density of 917 kg m^−3^ is used for ice, whereas for snow and firn, the density is measured in situ. In recent years, a network of 14 mass balance poles has been set up, distributed at regular distances along the central flowline of the glacier, which is about 25 km long and covers an elevation range of ca. 75 to 750 m a.s.l. The resulting point balances are used to establish a relationship with elevation, which is then used for extrapolation across the entire glacier surface (~100 km^2^). Comparison with geodetically derived volume changes (49) revealed good agreement along the centerline of Kongsvegen, but suggests that the glaciological method may overestimate the glacier-wide balance due to other influences on spatial pattern than elevation alone. Maintenance and survey of a mass balance network on a large, Arctic glacier requires considerable logistical efforts using snowmobiles in winter and helicopter in summer to cover the involved distances. Similar measurements are conducted at several glaciers across Svalbard, but only 12 of these have continuous records covering more than 5 y (11).

### CMB from Glacier–Climate Simulations.

For simulations of the CMB and runoff, we use the CryoGrid community model (19, 20), an open-source model developed for climate-driven simulations of the terrestrial cryosphere. It is a full energy-balance model with a modular structure, where different modules can be combined to describe the evolution of subsurface temperature, water content, and density under a wide range of surface and subsurface conditions. Here, a glacier (ice) module and a snow/firn module based on the CROCUS model (50) are used.

The model is forced by meteorological fields of 2 m air temperature, specific humidity, incoming long- and short-wave radiation, pressure, and mass fluxes. For this study, these are obtained from the Copernicus Arctic Regional ReAnalysis (CARRA) dataset (17, 51). CARRA is a high-resolution (2.5 × 2.5 km) reanalysis product over the European Arctic. It has been shown that CARRA has improved general verification statistics for all simulated regions compared to ERA5, with the largest differences associated with complex terrain (52). The dataset is updated monthly and currently spans the time frame from September 1990 to October 2024.

We use the same model setup and forcing as described in detail before (20), but for 2024, we use an updated glacier mask (14). We refer to this paper for further details on the model as well as validation of the 1991–2022 simulations.

The uncertainty of CryoGrid CMB simulations has been determined by (20), finding a RMSE of 0.39 m w.e. for the period 1991–2018, based on point measurements of glacier mass balance. However, model performance increased toward the end of the period (RMSE of 0.33 m w.e.), which is also noticeable in *SI Appendix*, Fig. S4*A*). We attribute the transient performance to either deficiencies in the spin-up of the model, lower skill of the CARRA reanalysis in a more data-sparse period (prior to 1997, see https://confluence.ecmwf.int/display/CKB/Copernicus+Arctic+Regional+Reanalysis+%28CARRA%29%3A+known+issues+and+uncertainty+information), or a combination of both.

This uncertainty is comparable to that of spaceborne elevation changes, specified by (53) to 0.35 m. For the land-terminating glaciers of Svalbard, both CMB and satellite-derived mass changes agree well within their uncertainty ranges (Fig. 4 of ref. 20), and again indicate better model performance toward the end of the period. To assign an uncertainty to our CMB estimates for 2024 (*SI Appendix*, Table S1), we use the RMSE of 0.33 m w.e. determined by (20) for 2016–2018. Applying this value to the entire glacier area yields mass change uncertainties of ±10.7 Gt (Svalbard) and ±3.9 Gt (Franz Josef Land) and ±6.6 Gt (Novaya Zemlya). In *SI Appendix*, Table S2 of ref. 20, the uncertainty of Kongsvegen CMB is close to the total uncertainty; our updated analysis for Kongsvegen (Fig. 1*C*) shows considerably smaller uncertainty for 2024. We argue that our uncertainty estimate for CMB in 2024 therefore represents a conservative estimate and the actual uncertainty presumably is considerably smaller.

### FA from Satellite Data.

FA is the sum of melting and calving at the marine glacier fronts and was estimated with established satellite-based methodology for Svalbard (22). First, area change of all ~200 marine-terminating glaciers was estimated by intersecting glacier frontlines from autumn 2024 with autumn 2023, using a Svalbard-wide annual dataset digitized from Sentinel-2 imagery (54). To account for ice discharge at the front, we defined a flux-gate line for each glacier 200 m inland of the most retreated front position. Glacier flow velocities were then extracted for each flux-gate line, resampled to a vertex point spacing of maximum 50 m. This was done at monthly time steps using the median velocity vector of the stacked ITSLIVE surface velocity product (55), in total ~4,000 velocity maps derived from image pairs with >5 d and <30 d separation.

Bedrock topography was obtained from the SVIFT 1.1 dataset (10) and differenced with ArcticDEM strip data (56) of surface elevation to estimate ice thickness for all area-change polygons and flux-gate lines. We used the median elevation of all ~900 DEM strips available from the year 2023, and at locations with fewer than five overlapping DEMs, we extended with 2022 and 2021 data. Areas without bedrock data were filled by interpolation between the glacier bedrock model and nearest ocean bathymetry data from IBCAO-v4.0 (57) and/or the NP S0 DEM (58) over land areas. Solid ice discharge for all glaciers in 2024 was then estimated by integrating the monthly velocity vectors with ice thickness along all flux gates, and then converted to mass by assuming an ice density of 917 kg m^−3^. Correspondingly, all frontal area changes were multiplied with ice thickness and density to account for mass changes due to frontal retreat or advance. The combined mass terms of ice discharge and front-change for all glaciers then give an estimate of total FA.

Uncertainties in surface velocity, elevation, and front position can be large locally, but reduce at the regional scale since they are of random nature. However, bedrock elevation under the glacier fronts is only in rare cases directly measured, i.e., where ice-penetrating radar data are available or when a glacier has advanced over an area with bathymetric survey data. Elsewhere, bedrock elevation is based on ice thickness modeling or interpolation between land-masked DEMs and bathymetry models, which can be prone to biases. According to (10), the mean calving front thickness on Svalbard is in the range 123 m to 158 m, which translates into a ~25% uncertainty span that scales directly with FA. Using this estimate and assuming that observational errors from area change, surface elevation, frontal velocity, and density each span up to 10% and are independent, we can estimate the overall uncertainty of FA from the root-sum-squared of these five estimates, yielding an uncertainty span of 32 or ±16% of the total FA estimate.

### TMB and Sea-Level Contribution.

The TMB is the sum of CMB and FA. To assess the TMB contribution to global sea-level change, we also must consider changes in submarine glacier volume (∆V_submarine_) which in case of glacier advance contributes to sea-level rise and in case of retreat to sea-level fall. We calculate this correction by multiplying the front-area change polygons with corresponding bedrock heights below sea level (water depth), obtaining a ∆V_submarine_ value of −3.2 km^3^ for 2024 (*SI Appendix*, Table S1). The sea-level equivalent (SLE) of the TMB (TMB = CMB + FA) is then:

$$TMB_{SLE}=-(CMB+FA-\Delta V_{\mathrm{submarine}})/A_{\mathrm{ocean}},$$

where A_ocean_ is the global ocean area (362.5 × 10^6^ km^2^), CMB and FA have been converted to volumetric units assuming a freshwater density of 1,000 kg m^−3^. TMB uncertainty is estimated from the root-sum-squared of the CMB and FA uncertainties, and we assume that the additional uncertainty in the conversion to SLE unit is negligible. For circum-Barents mass balance uncertainties, we take the sum of all regional uncertainties, in case they are correlated.

### Geodetic Constraints on Ice Mass Variations.

The Global Navigation Satellite System (GNSS) permits measuring the elastic response of the Earth’s crust to changes in surface loading (59). Mass changes of snow and ice represent a transient loading with significance at the Geodetic Earth Observatories in Svalbard (locations marked in Fig. 1*D*). GNSS records have been processed as described in (27, 60) using the GAMIT/GLOBK software (61), together with a global network of GNSS stations to ensure a global realization in ITRF2020 (62). The time series of vertical motions were corrected for a so-called common mode bias (63) using records from the station at Bjørnøya (BJOS). The annual uplift series, smoothed by a 60-d moving average, are presented in *SI Appendix*, Fig. S5.

### Temperature Observations and Weather Classification.

The three-decade span from 1991 to 2020 represents the most recent standard reference baseline period established by the World Meteorological Organization and was used as a baseline in this study. It also covers the period of the Arctic Regional reanalysis CARRA (51, 52). Since 1991, automatic weather stations on the northern and eastern islands of Svalbard have been monitoring Surface Air Temperature. Quality-controlled records (12) since 1991 are available for three stations: Edgeøya, Verlegenhuken, and Karl XII-øya. Additionally, data quality has been assessed for two other stations, Kvitøya and Kongsøya. Previous analyses (64–66) have already scrutinized and homogenized long-term daily temperature records from western Svalbard (Svalbard Airport and Ny-Ålesund), as well as Hopen in the southeastern region. The Polish Polar Station Hornsund (67) in south-western Svalbard provides a record dating back to 1978. All weather stations are located near the coast at elevations ranging from 5 to 28 m above sea level, making them susceptible to the influences of oceanic and sea-ice conditions throughout the year. Among these, Svalbard Airport and Ny-Ålesund are considered the most “continental,” situated within the fjords of Isfjorden and Kongsfjorden, respectively.

Analysis of air mass characteristics was based on an updated, large-scale atmospheric circulation type classification (AC) over western Svalbard (68) and previous analyses (37). This classification comprises 21 distinct AC types, represented by uppercase letters indicating the direction of air advection (for instance, N for northern and NE for northeastern) and lowercase letters denoting the nature of the pressure system (a for anticyclone and c for cyclone). Each day has been assigned to one of these 21 AC types. The air advection is aligned with the geostrophic wind direction, which is determined by the sea-level pressure pattern. In addition to the 16 types characterized by specific air advection, the classification also features four nonadvectional types (Ca for anticyclonic center over or near Spitsbergen, Ka for anticyclonic ridge, Cc for cyclone center over or near Spitsbergen, and Bc for cyclonic trough) along with one unclassified type, denoted as x.

### Empirical–Statistical Downscaling of CMIP6 Projections.

The empirical–statistical downscaling used the ERA5 reanalysis and 46 in situ thermometer measurements around the Barents region for the calibration of the downscaling method. The period 1956–2020 was used for calibrating the downscaling method which used common empirical orthogonal functions as a framework to ensure a good match between the calibration and results from the global climate models (69), and the downscaling strategy was the same as used in a previous study (44). It was carried out in the R-environment (https://www.r-project.org/) and used the R-package “esd,” which is freely available from https://github.com/metno/esd. The predictand representing the local in situ temperature is based on the seasonal mean temperature, and principal component analysis was used to represent the group of sites surrounding the Barents Sea (70), and since the aggregated daily temperature was approximately normally distributed, a stepwise ordinary linear multiple regression was used to train the downscaling method. The performance of the downscaling method was evaluated both in terms of traditional cross-validation and when applied to global climate model results (44).

## Supplementary Material

Appendix 01 (PDF)

## Data Availability

The C3S Arctic Regional Reanalysis CARRA is available at the Copernicus Climate Change Service (C3S) Climate Data Store (CDS) (71) https://cds.climate.copernicus.eu/. A surface elevation model (58) and updated glacier outlines for Svalbard (14, 54) are available at the Norwegian Polar Data Centre https://data.npolar.no/. Cryogrid simulations for Svalbard (72) and Franz Josef Land and Novaya Zemlya (73) are available at the Arctic Data Centre https://adc.met.no. AWS data from MET-Norway are freely available from https://seklima.met.no/days/mean(air_temperature%20P1D)/custom_period/SN99840,SN99735,SN99720,SN99754,SN99935,SN99740,SN99938,SN99910,SN99927/nb/1991-01-01T00:00:00+01:00;2024-11-01T23:59:59+01:00. Glacier-wide mass balances for Kongsvegen are available in the database of the World Glacier Monitoring Service (https://doi.org/10.5904/wgms-fog-2024-01) and at Environmental Monitoring of Svalbard and Jan Mayen https://mosj.no/en/indikator/climate/land/mass-balance-for-glaciers-in-svalbard/. ITS_LIVE glacier velocities have been obtained from ref. 55, ArcticDEM strip data from ref. 56, and the IBCAO bathymetry from ref. 57. The ice-free topography of Svalbard is available from ref. 10. Daily GNSS land uplift rates for 2024 and mean, min, max for 2010–2020 are available from ref. 74. The CryoGrid model (19) is available at https://github.com/CryoGrid/CryoGridCommunity_source and Zenodo https://doi.org/10.5281/zenodo.6522424. Code for the empirical–statistical downscaling is available at https://github.com/metno/esd.
