# Supplementary material for: Svalbard’s 2024 record summer: An early view of Arctic glacier meltdown?
Source: Proc Natl Acad Sci U S A. 2025 Aug 18;122(34):e2503806122. doi: 10.1073/pnas.2503806122 (PMC12403128; doi:10.1073/pnas.2503806122)
Supplement: Supplementary file 1 — Appendix 01 (PDF) [file pnas.2503806122.sapp.pdf]

Supporting Information for

Svalbard's 2024 Record Summer: An Early View of Arctic Glacier Meltdown?

Thomas Vikhamar Schuler *, Rasmus Emil Benestad, Ketil Isaksen, Halfdan Pascal Kierulf, Jack Kohler, Geir Moholdt, Louise Steffensen Schmidt

* corresponding author: Thomas V Schuler
Email: t.v.schuler@geo.uio.no

This PDF file includes:

Table S1
Figures S1 to S11
SI References

Table S1: Mass balance components and sea-level contribution.

Mass balance components (CMB, frontal ablation) and sea-level impact (after correction for submarine volume change) for the three glacier regions of Svalbard, Franz Josef Land and Novaya Zemlya in the mass balance year 2023/24, together accounting for the total circum-Barents glacier region.

Region	climatic mass balance, CMB (Gt)	frontal ablation, FA (Gt)	total mass balance, TMB (Gt)	submarine volume change (km^3)	sea-level contribution (km^3)	sea-level equivalent (mm)
Svalbard	-42.1±10.7	-19.6±3.1	-61.7±11.1	-3.2±0.5	58.5±11.1	0.16±0.03
Franz Josef Land	-7.8±3.9	-7.4±3.3 [a]	-15.2±5.1	-1.7±1.8 [a]	13.5±5.4	0.04±0.01
Novaya Zemlya	-21.1±6.6	-4.2±0.9 [a]	-25.3±6.7	-0.7±0.6 [a]	24.6±6.7	0.07±0.02
Circum-Barents region	-71.0±21.2	-31.2±7.3	-102.2±22.9	-5.6±2.9	96.6±22.9	0.27±0.06

[a] decadal 2010-2020 rates of frontal ablation and submarine volume change (1)

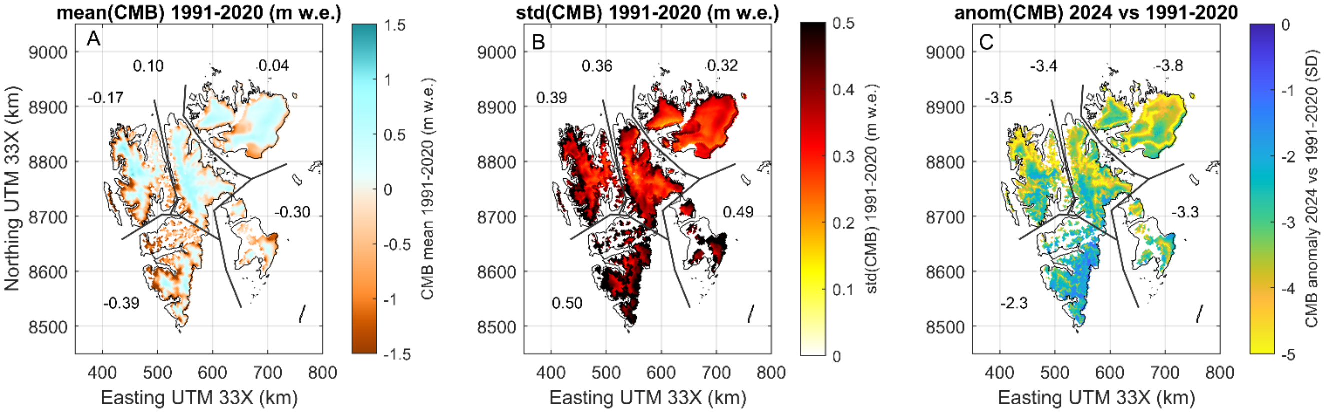

Figure S1. Spatial patterns of CMB, its variability and the 2024 anomaly
(A) Area-specific, mean CMB for the 1991-2020 climatology, expressed in m w.e.; boundaries between subregions are shown in black and labels indicate values for each region (m w.e.)
(B) Year-to-year variability of CMB for the 1991-2020 climatology, represented by standard deviation (std; m w.e.).
(C) Normalized anomaly of CMB in 2024 vs the 1991-2020 climatology, expressed in terms of standard deviation.
The 2024 CMB anomaly was less pronounced in southern Spitsbergen, even though it was still at a record level. Southern Spitsbergen in general is warmer and has higher summer melt rates and being closer to the threshold between melting and freezing, the CMB variability is larger compared to the colder regions further north.

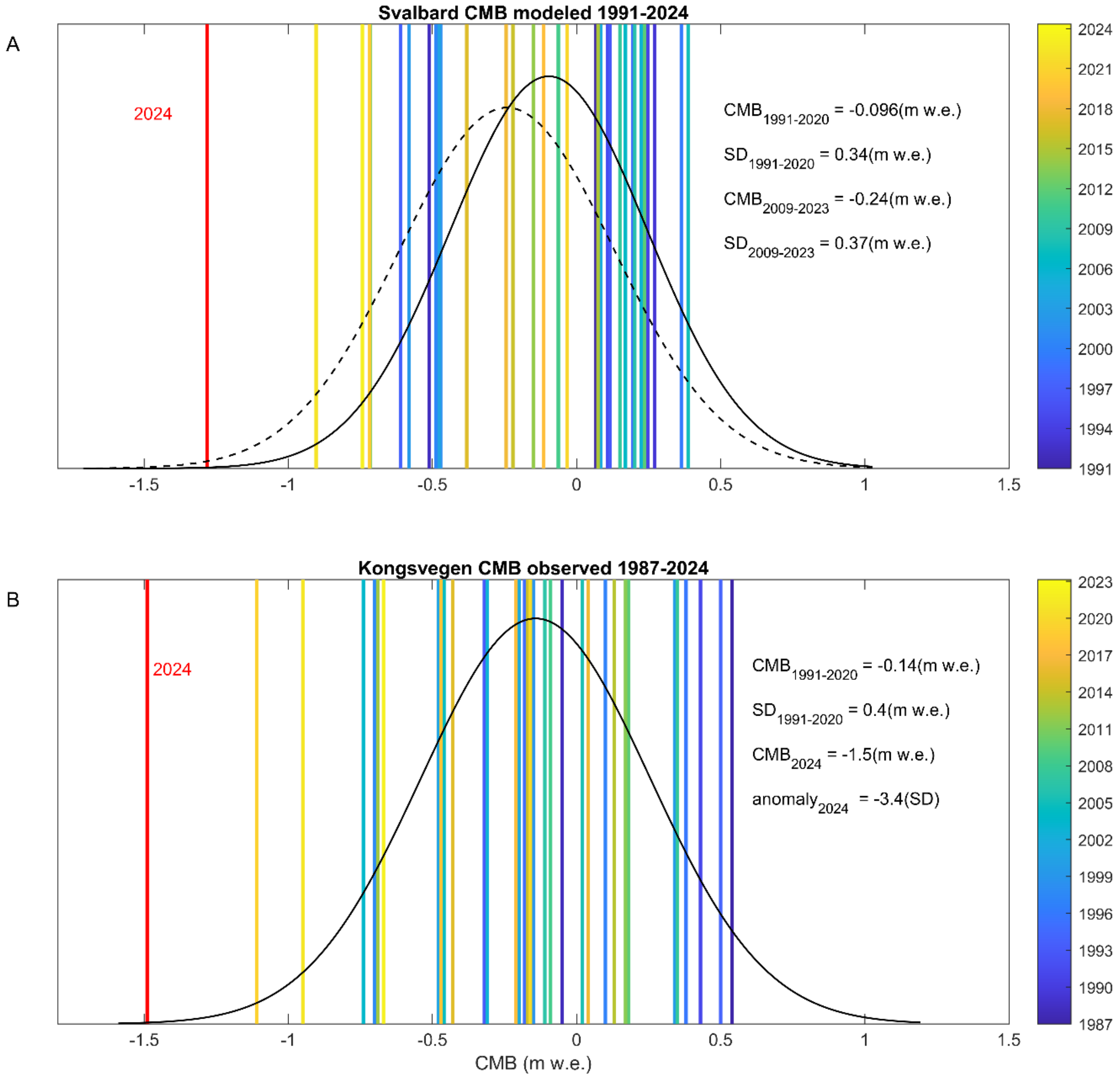

Figure S2. Statistical distribution of glacier mass balance
(A) modeled Svalbard-wide CMB 1991-2024;
(B) observed Kongsvegen glacier-wide mass balance 1987-2024.
The vertical lines denote individual values and are color-coded to indicate year; the red line shows 2024, and the black solid line is a normal distribution fitted to the 1991-2020 climatology. One may argue that the climatology 1991-2020 is non-stationary, and as a quick alternative evaluation, we consider only the more recent 15-years period 2009-2023 when CMB was more negative (black, dashed line in (A). Although area-specific values for a single glacier are not representative for the entire population of glaciers in Svalbard (consisting of 2010 individual glaciers (2)), temporal variations are similar, resulting in comparable characteristics in terms of anomaly and return interval of the 2023/24 conditions.

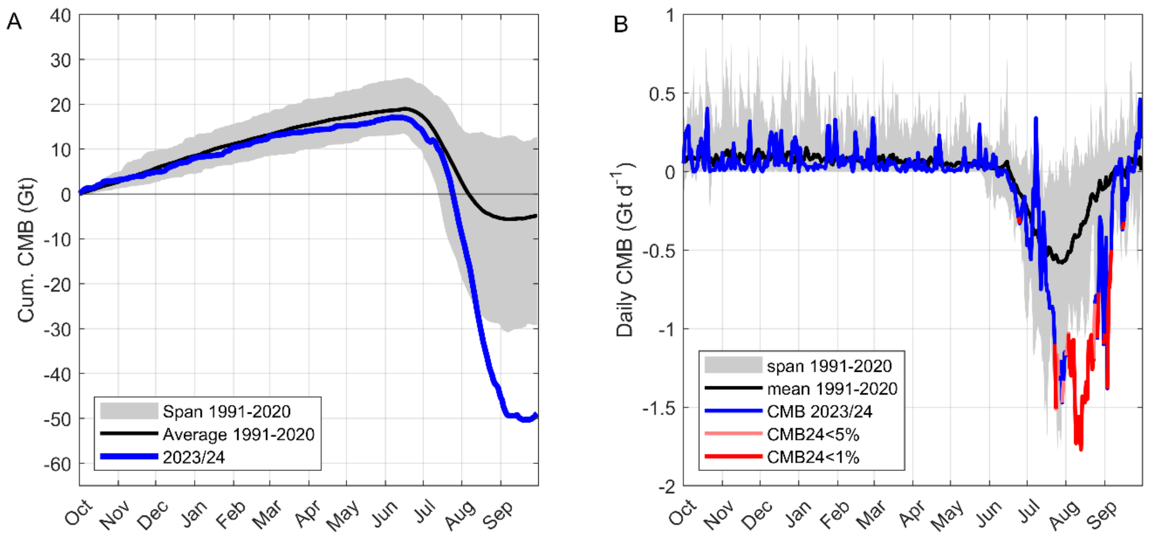

Figure S3. Comparison of 2024 CMB vs the 1991-2020 CMB climatology
(A) Cumulative CMB for all Svalbard glaciers for the season 2023/24 (blue), compared to the mean (black) and range of the 1991-2020 climatology (gray).
(B) Daily CMB (GT) for all Svalbard glaciers for the season 2023/24 (blue), compared to the mean (black) and range of the 1991-2020 climatology (gray). Red colors indicate where the 2024 CMB was below the 5% (1%) percentiles of the 1991-2020 climatology.

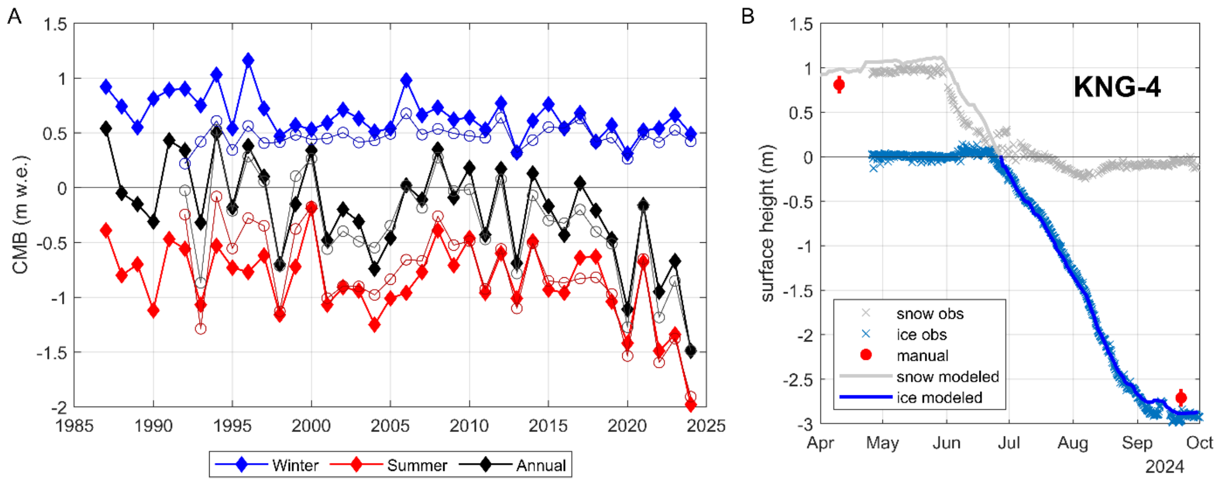

Figure S4. Model performance during the extreme year 2024
(A) Measured and modeled glacier-wide mass balance of Kongsvegen glacier. Observed values are shown with filled diamonds and open circles refer to modelled values. The annual balance (Oct preceding year-Sep current year, black) and seasonal components, winter (Oct-Apr, blue) and summer (May-Sep, red). The agreement between observations and simulation is evaluated in Fig 1 C, including the balance year 2023/24.
(B) Measured and modeled surface height evolution of Kongsvegen glacier at ~380 m a.s.l.. Simulated surface height evolution during the summer of 2024 (solid line) compared with measurements of snow depth (gray) and ice surface height change (blue) using an autonomously recording device (3). Markers denote measured values, whereas the solid line refers to the simulation. The red symbols mark manual measurements relative to a mass balance stake fixed into the ice.

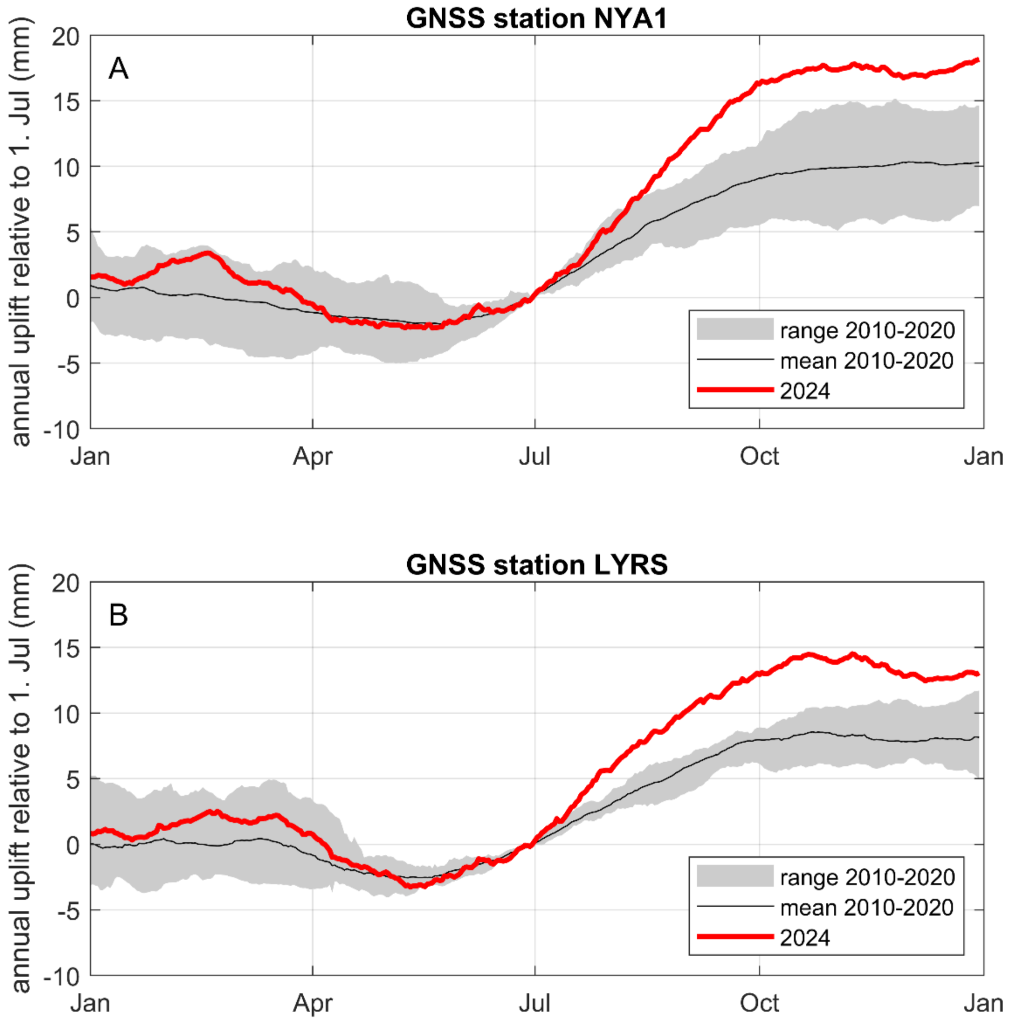

Figure S5. Annual uplift measured at the Svalbard Geodetic Earth Observatories. (A) NYA1 at Ny Ålesund and (B) LYRS at Longyearbyen, the locations of which are marked in Fig. 1. To visualize the uplift during the summer period (1. Jul - 1. Oct), annual uplift values are displayed relative to 1. July.

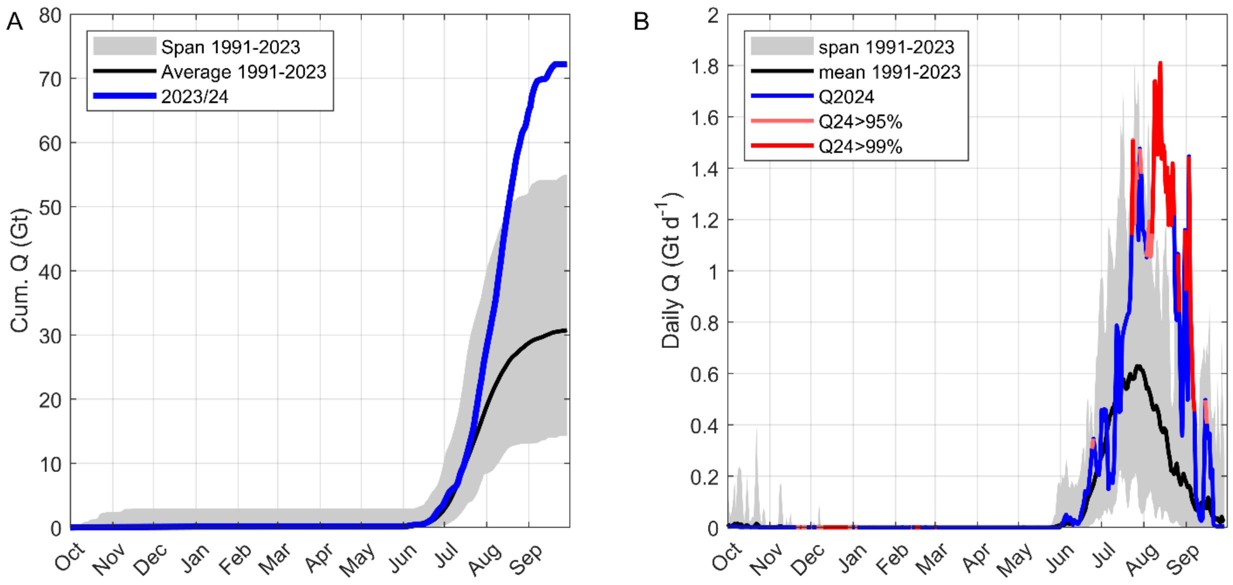

Figure S6. Comparison of 2024 runoff levels vs 1991-2020 runoff climatology
(A) Cumulative runoff from Svalbard for the period Oct 2023 - Oct 2024. The gray shading indicates the range of values in preceding years 1991-2023, the black line denotes the average for the same period and the blue line shows the current year.
(B) Daily runoff from Svalbard for the period Oct 2023 - Oct 2024. The gray shading indicates the range of values in preceding years 1991-2023, the black line denotes the average for the same period and the blue line shows the current year.

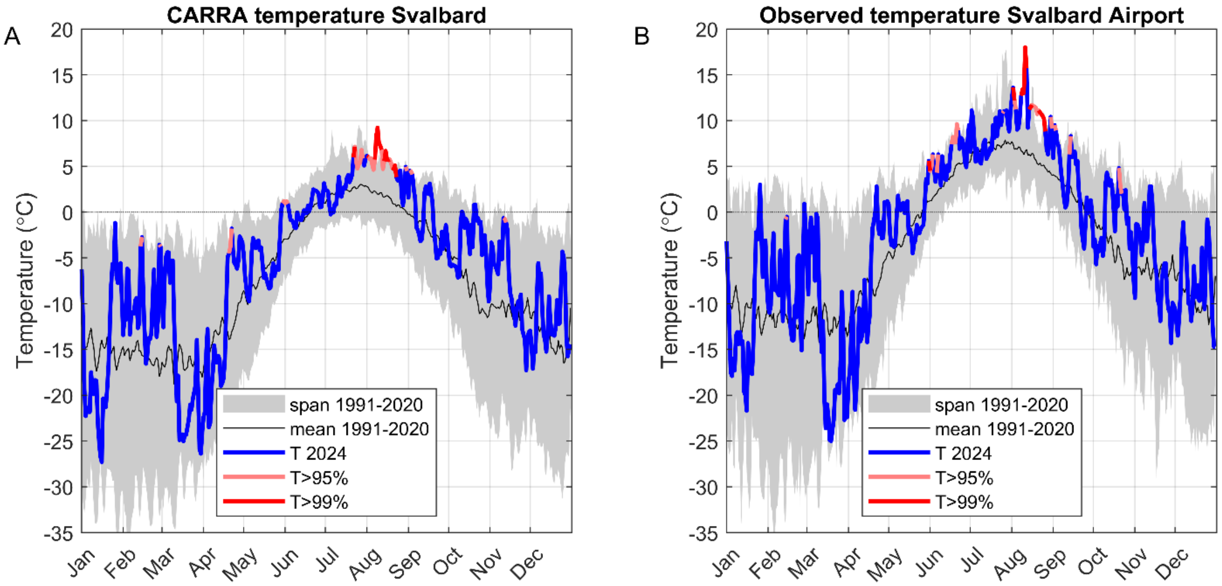

Figure S7. Comparison of 2024 air temperature vs 1991-2020 temperature climatology
Daily surface air temperature in 2024 (blue) compared to the range of values for the 1991-2020 climatology (gray) and its mean (black); pink (red) indicates when the temperature in 2024 exceeded the 95% (99%) percentile of the 1991-2020 climatology:
(A) Svalbard-wide temperature from CARRA reanalysis,
(B) observed temperature record from Svalbard Airport weather station.

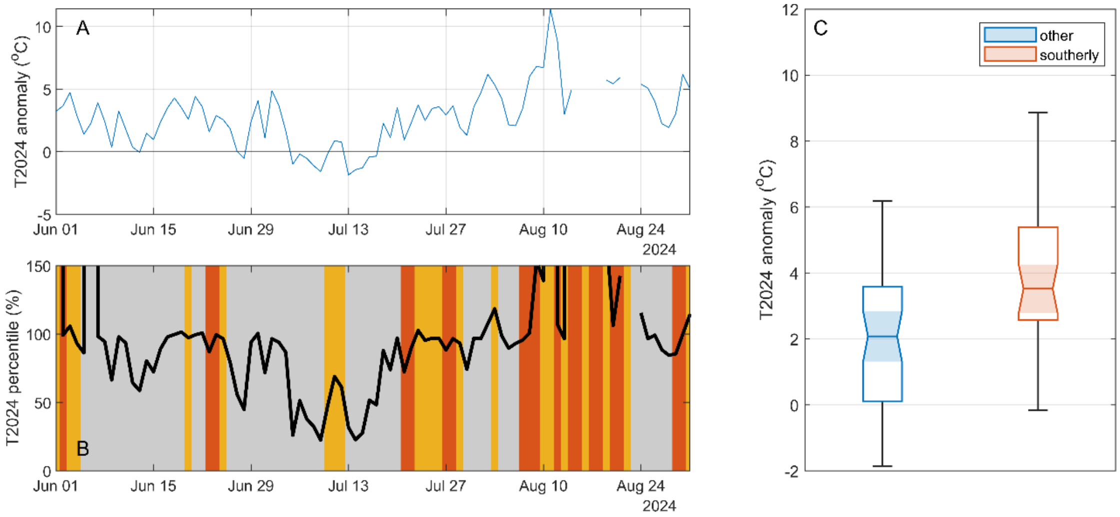

Figure S8. (A) summer T-anomalies and (B) the percentiles of the anomalies compared to the 1991-2020 climatology, along with the occurrence of circulation types (S, SW-SE, others). High T-anomalies also occur for other circulation directions, probably explained by the inertia of the system. (C) boxplots of the T-anomalies grouped for other and southerly circulation types showing clear temperature differences. The notches of the two categories do not overlap, indicating that the median temperature anomalies for the southerly (+3.52 °C) and other (+1.84 °C) circulation types are significantly different at the 5% level.

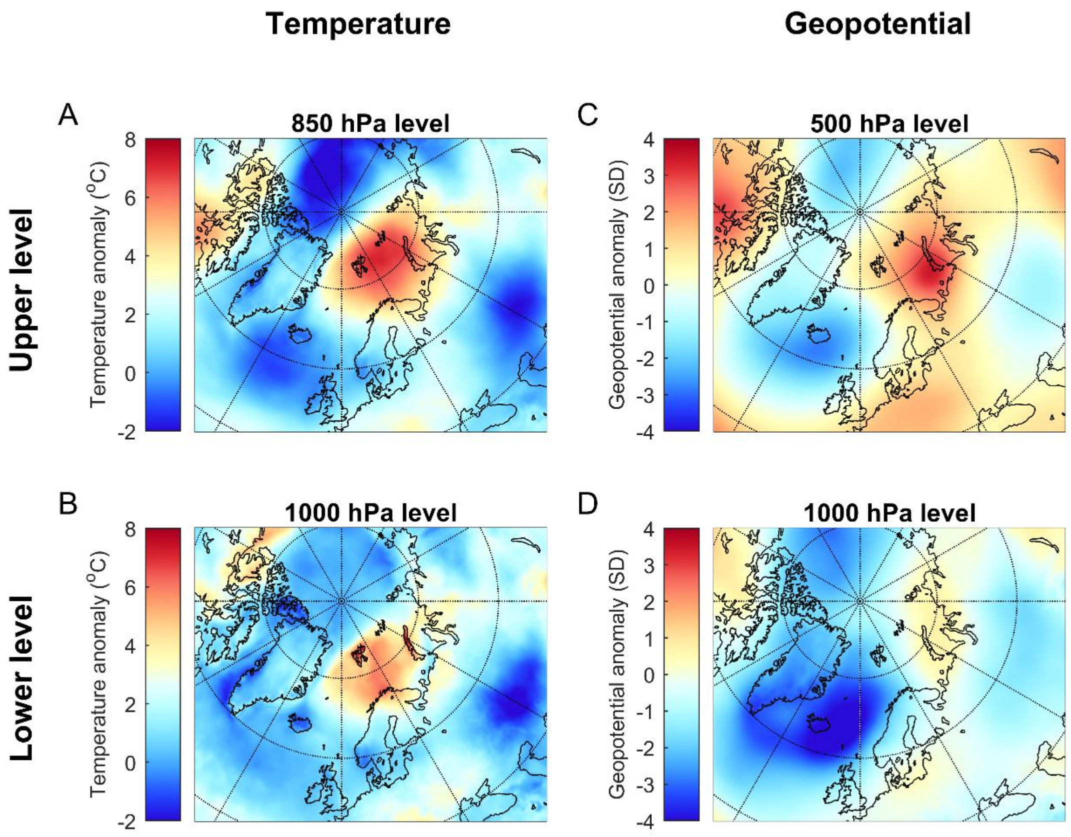

Figure S9. ERA-5 (4) anomalies of temperature (A, B) and geopotential (C, D) during August 2024 with respect to the 1991-2020 reference period. (A) and (C) show anomalies in upper pressure levels of the troposphere, and (B) and (D) close to the surface. Temperature at 850 hPa and geopotential at 500 hPa are frequently used to characterize circulation patterns (5). The positive anomalies in both cases are more pronounced for the upper levels, suggesting that the anomalous surface air temperature in Svalbard (Fig. 3) was not only a surface phenomenon.

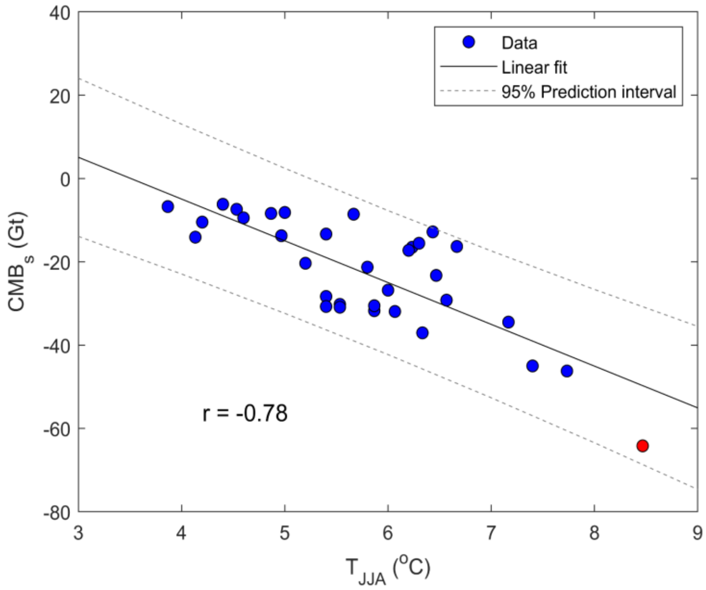

Figure S10. Relationship between Svalbard summer balance and summer air temperature. Values for the period 1991-2024, with 2024 value highlighted (red). The solid line shows a linear fit, along with 95% prediction interval (dashed). The correlation (r=-0.78) is similar to that found by (6), but the scatter clearly indicates that CMB_s also depends on other factors than just temperature (among others depending on the previous winter season, cloudiness etc).

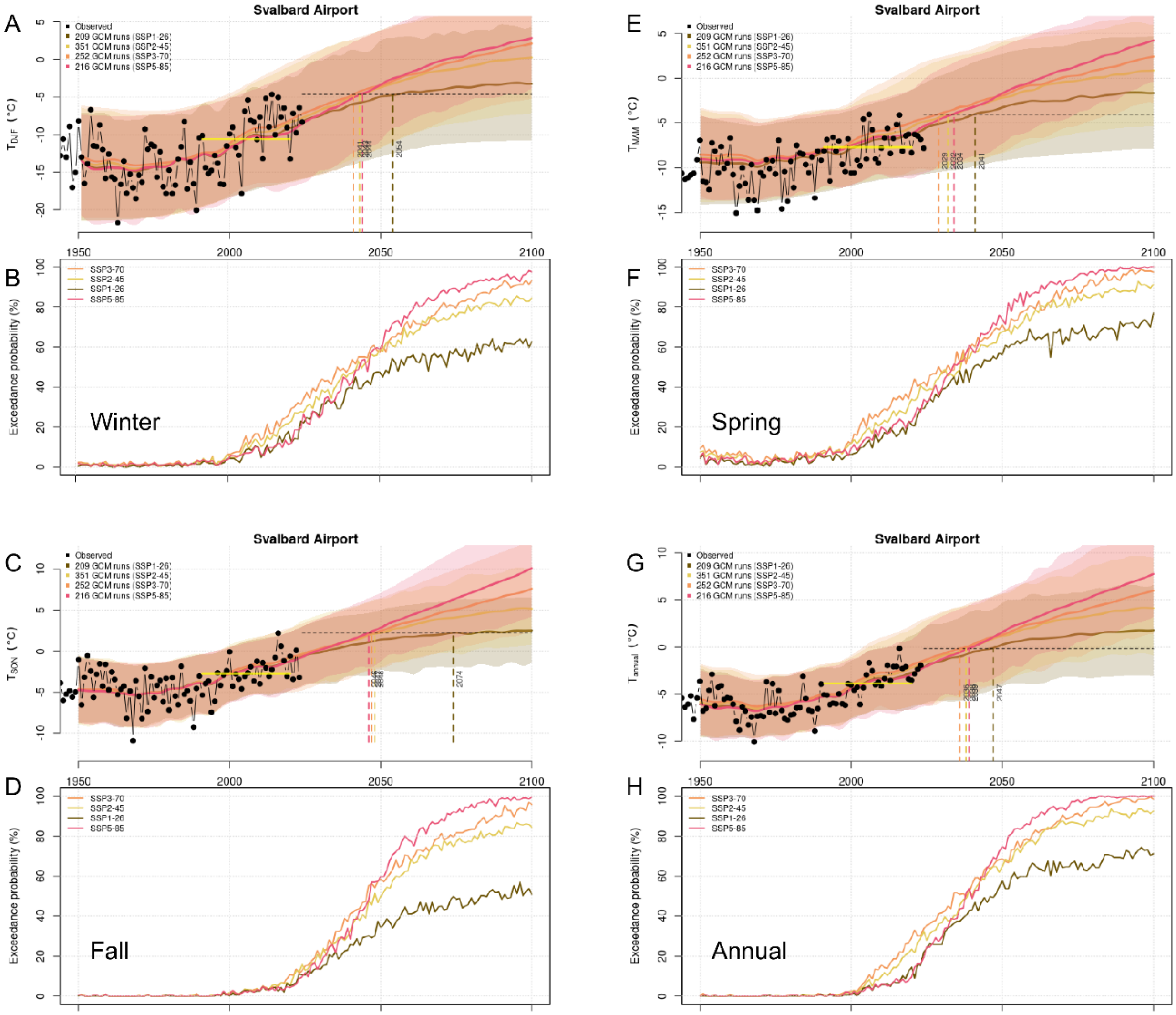

Figure S11. Projected temperature evolution until 2100
Equivalent to Fig 4 but for the other seasons, winter DJF (A, B), spring MAM (E, F) and fall SON (C, D) as well as for the annual (G, H) mean surface temperature at Svalbard Airport, along with probability of exceeding the observed record level for each of the four SSP ensembles.

SI References

1. Ø. Foss, *et al.*, Ocean warming drives immediate mass loss from calving glaciers in the high Arctic. *Nat Commun* **15**, 10460 (2024).

2. J. Kohler, A. Lith, G. Moholdt, J. Kohler, Svalbard glacier inventory based on Sentinel-2 imagery from summer 2020. Norwegian Polar Institute. https://doi.org/10.21334/NPOLAR.2021.1B8631BF. Deposited 2021.

3. R. S. Fausto, D. Van As, A. P. Ahlstrøm, M. Citterio, Assessing the accuracy of Greenland ice sheet ice ablation measurements by pressure transducer. *J. Glaciol.* **58**, 1144–1150 (2012).

4. Copernicus Climate Change Service, ERA5 monthly averaged data on pressure levels from 1940 to present. Copernicus Climate Change Service (C3S) Climate Data Store (CDS). https://doi.org/10.24381/CDS.6860A573. Deposited 2019.

5. M. Tedesco, X. Fettweis, Unprecedented atmospheric conditions (1948–2019) drive the 2019 exceptional melting season over the Greenland ice sheet. *The Cryosphere* **14**, 1209–1223 (2020).

6. E. C. Geyman, W. J. J. Van Pelt, A. C. Maloof, H. F. Aas, J. Kohler, Historical glacier change on Svalbard predicts doubling of mass loss by 2100. *Nature* **601**, 374–379 (2022).
